# Gastric Duplication: A Rare Cause of Massive Lower Gastrointestinal Haemorrhage, Chest Wall Mass, and Enterocutaneous Fistula

**DOI:** 10.1155/2012/250890

**Published:** 2012-08-26

**Authors:** Emeka B. Kesieme, Andrew E. Dongo, Clement O. Osime, Sylvia C. Olomu, Oluwafemi O. Awe, Gerald I. Eze, Sylvester U. Eluehike

**Affiliations:** ^1^Department of Surgery, Irrua Specialist Teaching Hospital, PMB 8, Irrua, Nigeria; ^2^Department of Paediatrics, Irrua Specialist Teaching Hospital, PMB 8, Irrua, Nigeria; ^3^Department of Pathology, Irrua Specialist Teaching Hospital, PMB 8, Irrua, Nigeria; ^4^Department of Radiology, Irrua Specialist Teaching Hospital, PMB 8, Irrua, Nigeria

## Abstract

Gastric duplications are uncommon developmental abnormality reported to present with different clinical scenarios. We present a 2-1/2-year-old Nigerian female who started having intermittent massive lower gastrointestinal haemorrhage at 5 months of age. She subsequently developed a lower chest wall mass and enterocutaneous fistula. She was found to have gastric duplication with fistulous communication with the descending colon, spleen, and lower chest wall. To the best of our knowledge, this is the first paper on gastric duplication resulting in intermittent massive lower gastrointestinal bleeding mainly from splenic capsular erosion and fistula and enterocutaneous fistula resulting from erosion of anterior abdominal wall. Gastric duplication is hence an important rare cause of intermittent massive lower gastrointestinal haemorrhage and spontaneous enterocutaneous fistula in the paediatric population.

## 1. Introduction 

Gastric duplications are rare congenital anomalies accounting for 4%-5% of all gastrointestinal malformation [1]. They are mainly seen during the first year of life and are typically more common in females.

The symptoms depend on the location. They are usually nonspecific or they may result secondary to complication.

We report a case of gastric duplication who presented with intermittent massive lower gastrointestinal bleeding and enterocutaneous fistula. A fistulous connection to descending colon, spleen, and lower anterior chest wall was discovered.

## 2. Case Report

The patient is a 2-1/2-year-old Nigerian female child who presented with recurrent massive lower gastrointestinal haemorrhage since 5 months of age, left-sided lower chest wall swelling of 9 months duration which was gradually increasing in size but later formed a sinus and started discharging mucopurulent and haemorrhagic effluent 2 days prior to presentation and chest pain of 1-year duration. On account of bleeding, the patient had been transfused severally in the past and she had an exploratory laparotomy in a tertiary hospital 8 months prior to presentation. However no abnormality was found by the operating surgeon. Her symptoms did not improve. There is no history of bleeding disorder in the family. Haemoglobin genotype is AA.

On examination at presentation, she was pale, not dyspneic, but she had a slightly tender left axillary lymphadenopathy. Vital signs were stable. There was an asymmetrical, ill-defined chest wall mass overlying the 9th and 10th ribs with a central sinus discharging mucoid and haemorrhagic fluid. The surrounding skin was erythematous and edematous. Examination of the abdomen revealed a transverse infraumbilical incision scar. No other abnormality was noted. A chest radiograph done suggested periosteal reaction involving the 9th and 10th ribs. Wound swab MCS, wound biopsy, and biopsy of 2 enlarged and discrete axillary lymph nodes were done. A wound swab culture grew *E. coli*. Acid fast bacilli test was negative and Mantoux test was not significant. The histology of the biopsied lymph nodes was in keeping with reactive hyperplasia. Packed cell volume (PCV) on admission was 27%.

**Figure 1:**
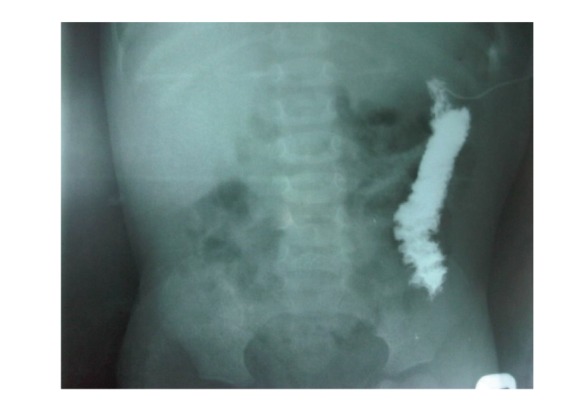
Fistulogram showing contrast emptying into the descending colon.

Three days on admission, faeces started discharging from the sinus on the left lower chest wall. She developed 3 episodes of massive lower gastrointestinal bleeding with bleeding per rectum and bleeding through the enterocutaneous fistula site. Packed cell volume dropped to 23% and 21% and patient was transfused to build up the PCV to 30%. Fistulograph done showed the free flow of contrast into the descending colon (Figure 1).

**Figure 2:**
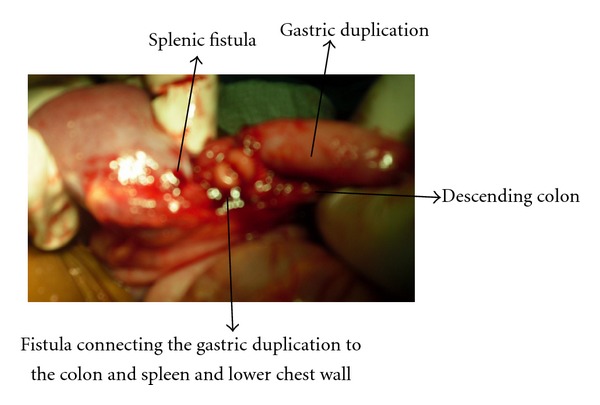
Common fistula connecting the gastric duplication with the descending colon, spleen (and splenic erosion), and the strand of pancreatic tissue.

**Figure 3:**
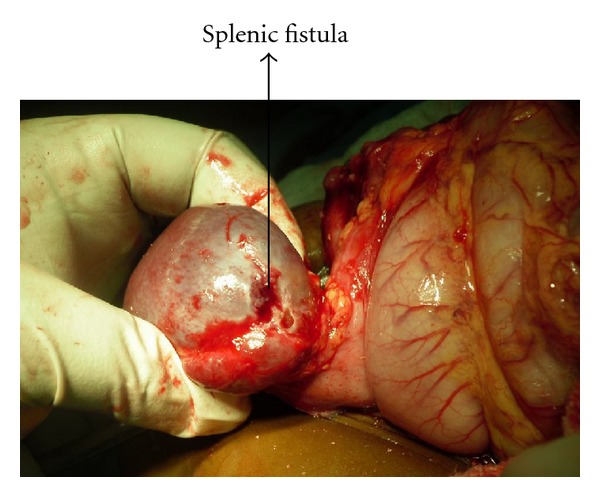
Splenic erosion and splenic fistula.

She was worked up for exploratory laparotomy. Intraoperative findings included gastric duplication measuring 4 × 5 cm, originating from the greater curvature. It did not communicate with the gastric lumen. There was a fibrous cordlike extension extending from the body of pancreas to the gastric duplication consisting principally of pancreatic tissue. The gastric duplication had a fistulous connection to the descending colon, measuring about 1.5 cm in diameter and the same fistulous tract also causing splenic capsular erosion and splenic fistula and a communication to the lower chest wall causing colocutaneous fistula. (Figures 2 and 3). She had excision of the gastric duplication, excision of the colonic fistula and devine colostomy. She is awaiting colostomy closure. She has been followed up in the outpatient clinic. She has no complaints and has gained weight considerably.

**Figure 4:**
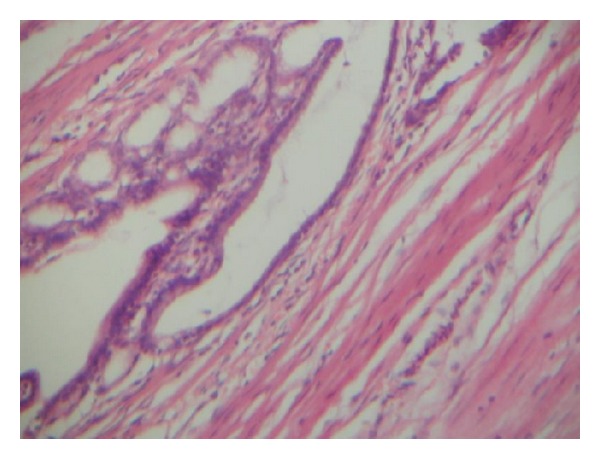
Gastric epithelium of the duplication (H&E ×40).

**Figure 5:**
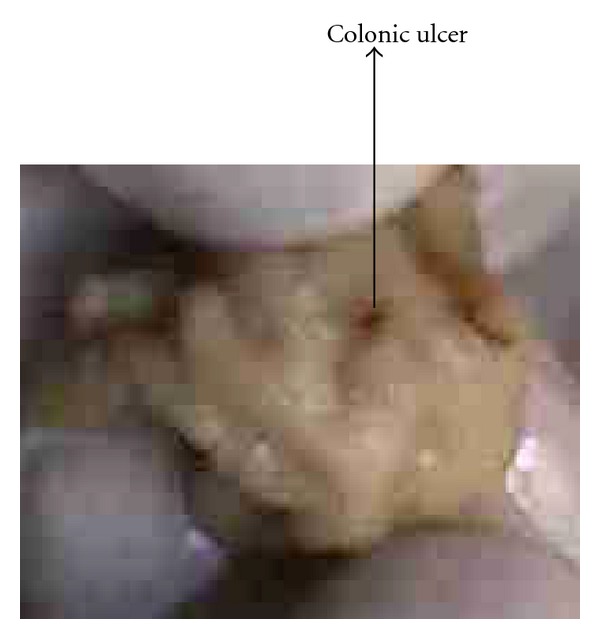
Colonic ulcer.

Histology of the specimen confirmed gastric epithelium (Figure 4), while the fibrous strand was confirmed histologically as being pancreatic tissue. A tiny colonic ulcer was found in colon adjacent to the fistula (Figure 5) while microscopic colonic ulcers were seen.

## 3. Discussion

Many theories have been postulated to account for the aetiology of gastric duplication, with none of them explaining the malformation completely. They include aberrant recanalization of the gastrointestinal tract, persistent embryological fetal gut diverticulum, and uteroischaemic events [2].

This case fulfilled the morphological criteria described by Ladd for gastrointestinal duplication, which include the presence of well developed smooth muscle, epithelial lining from the alimentary tract, and an attachment to some part of the alimentary tract [3]. The mucosal lining was gastric epithelium. This differed with previous reports in which heterotrophic pancreatic tissues were discovered in the gastric epithelial lining [4]. The presence of a fibrous cordlike extension principally consisting of pancreatic tissues was also reported by Rosenlund and Schnaufer; however, their case was asymptomatic [5].

Our patient developed intermittent massive rectal bleeding with the initial episode presenting at 5 months of age. Similar reports of massive lower gastrointestinal bleeding have been reported in a 5 months and 11 months old females [4, 6]. Both cases perforated through the transverse colon.

The source of bleeding in our patient was primarily from the splenic capsular erosion and fistula with contribution from colonic ulcers. This differs from the case reported by Mahnovski et al. [6], where the source of bleeding was entirely from the mucosal ulcer of the transverse colon opposite the opening of the fistula and the case reported by Costa et al. [4], in which case the haemorrhage was from the site of perforation.

Massive upper gastrointestinal haemorrhage has also been reported as a complication of gastric diverticulum [7].

Erosion of the lower chest wall was responsible for the significant chest wall soft-tissue edema, periostitis involving the 9th and 10th ribs, and discolouration of skin. The picture was initially confused to be a rib osteomyelitis or an unsuspected rib fracture. The case reported by Bonacci and Schlatter also reported chest and anterior abdominal wall edema, 7th rib periosteal changes and pleural effusion [7]. However in our case, erosion of the lower chest wall worsened to form a colocutaneous fistula. Erosion and fistulous connection to the spleen and enterocutaneous fistula have never been reported as complicating gastric duplication. Most of the reported cases of perforation into the intestine had either pancreatic attachment or contained heterotrophic pancreatic tissue [4, 5]. This substantiates the hypothesis that perforation may result from gastric acid secretion and inflammatory factors from pancreatic tissue. It has been argued that the latter has minimal or no contribution, since it is relatively small and discrete [7]. The splenic erosion and fistula could have also resulted from pancreatic enzymatic action.

The treatment modality depends on the presentation. Surgery is indicated in symptomatic or complicated cases.

## 4. Conclusion 

This case illustrates an unusual presentation of gastric duplication. Gastric duplication is a rare cause of recurrent massive lower gastrointestinal haemorrhage associated with lower chest wall enterocutaneous fistula.
